# Multifocal tDCS targeting lower-limb cortical areas preserves late-stage endurance and tunes phase-specific coordination during incremental cycling

**DOI:** 10.3389/fphys.2025.1706478

**Published:** 2025-12-02

**Authors:** Zhiqiang Liang, Perianen Ramasawmy, Xue Guo, Yufei Fang, Andrea Antal, Yu Liu

**Affiliations:** 1 Research Academy of Medicine Combining Sports, Ningbo No. 2 Hospital, Ningbo, China; 2 Faculty of Sports Science, Ningbo University, Ningbo, China; 3 Key Laboratory of Exercise and Health Sciences of Ministry of Education, Shanghai University of Sport, Shanghai, China; 4 Department of Neurology, University Medical Center Göttingen, Georg-August University, Göttingen, Germany; 5 Department of Rehabilitation Medicine, Ningbo No. 2 Hospital, Ningbo, China

**Keywords:** multifocal transcranial direct current stimulation, lower-limb cortical networks, incremental cycling test, endurance performance, muscle coordination

## Abstract

**Background:**

Multifocal transcranial direct current stimulation (m-tDCS) may modulate distributed motor networks in a polarity-dependent and task-state-dependent manner to support performance near exhaustion. To this end, this study aims to test whether m-tDCS targeting lower-limb-specific cortical areas could optimize late-stage performance and phase-specific muscle coordination during cycling.

**Methods:**

Two independent trials were conducted: (i) a tolerability assessment (Trial 1) and (ii) a randomized, double-blind, sham-controlled parallel study (Trial 2). In Trial 1, participants completed the tolerability test and recorded pain and side effects during a 21-min stimulation period. In Trial 2, healthy adults completed an incremental cycling test; late-stage performance was operationalized *a priori* as the 85%–100% peak power output (PPO) phase, during which the time-to-exhaustion (TTE), work (W), mean power (P), revolutions per minute (RPM), heart rate (HR), blood lactate level (ΔL), ratings of perceived exertion (RPE), and EMG-derived muscle contribution ratio (MCR) and knee co-activation index (CAI) were analyzed across propulsion and pull.

**Results:**

(1) m-tDCS was well-tolerated; pain ratings declined progressively across the stimulation, with typical transient sensations. (2) At the 85%–100% PPO phase, m-tDCS increased W and RPM relative to sham without altering the mean power or ΔL; HR decreased after m-tDCS, and RPE rose only after sham. (3) At the coordination level, m-tDCS preserved quadriceps MCR during propulsion and reduced antagonistic activation during pull to prevent the CAI increase observed in the sham.

**Conclusion:**

m-tDCS did not augment peak mechanical output but preserved late-stage endurance via phase-specific coordination tuning, which is consistent with improved neural efficiency near exhaustion. These findings refine mechanistic interpretations of the effects of tDCS on endurance and support m-tDCS as a safe, coordination-centric adjunct for high-intensity cycling.

## 1 Introduction

Endurance performance in cycling is influenced by the interplay between central drive, peripheral fatigue, and neuromuscular coordination, especially during the high-intensity terminal phases of exercise. A growing body of research indicates that non-invasive brain stimulation (NIBS) can exert promising ergogenic effects on motor capacity across diverse populations; however, these effects are modality- and protocol-dependent, with different modalities of NIBS producing distinct physiological responses.

Transcranial direct current stimulation (tDCS), one of the most widely used and safe NIBS techniques in sports science and rehabilitation, modulates cortical excitability and network dynamics in a polarity-specific and state-dependent manner. Its ergogenic effects are confirmed to be gated by the ongoing “task state,” such that the same current can yield different behavioral consequences at rest compared with during an engaged motor task (Nitsche and Paulus, 2000; Stagg and Nitsche, 2011; Silvanto et al., 2008; Polanía et al., 2018). In contrast, transcranial magnetic stimulation (TMS), another NIBS technique, delivers brief, focal magnetic pulses that directly evoke action potentials; it is also commonly used to probe corticospinal function and central fatigue rather than as an on-task ergogenic intervention (Hallett, 2007; Lefaucheur et al., 2020). Thus, TMS should be considered a tool to provide complementary mechanistic benchmarks, whereas tDCS may offer a practical means to optimize motor control during fatiguing activity (Nitsche and Paulus, 2000; Nitsche and Paulus, 2001; Stagg and Nitsche, 2011; Polanía et al., 2018).

However, evidence supporting the beneficial effects of tDCS on exercise performance remains mixed: some studies report improvements in endurance performance and perceived exertion (e.g., longer time to exhaustion or reduced perceived effort) (Vitor-Costa et al., 2015; Okano et al., 2015; Angius et al., 2016), whereas meta-analyses indicate generally small, protocol-sensitive effects with substantial heterogeneity across tasks (Machado et al., 2019; Holgado et al., 2019; Kaushalya et al., 2022; Holgado et al., 2024; Flor et al., 2025). One approach to reduce variability and enhance functional specificity is to use multifocal electrode montages that target distributed motor networks rather than a single scalp site; both modeling work and empirical work suggest that multi-electrode configurations can improve focality and effective intensity at the target while engaging interconnected cortical regions (Dmochowski et al., 2011; Huang and Parra, 2019).

Cycling performance requires phase-specific coordination of muscle synergies, with task-relevant contributions during the propulsion phase and potential antagonistic activation during the pull phase. Electromyographic studies have demonstrated robust muscle synergy structures and rate-dependent coordination (Neptune et al., 1997; Hug and Dorel, 2009; Hug et al., 2011). From this perspective, indices such as the muscle contribution ratio (MCR) and co-activation index (CAI) provide concise and interpretable summaries of how neuromuscular effort is distributed across different phases, capturing both task-relevant activation and the inhibition of non-task-related co-activation.

**FIGURE 1:**
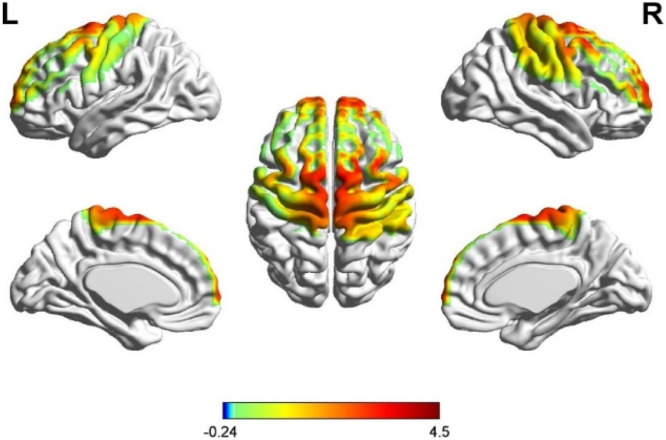
Activation of prefrontal and motor cortices at the termination phase.

Moreover, growing evidence suggests that prefrontal cortical mechanisms play a significant role in regulating effort and terminating tasks during endurance exercise (Qi et al., 2024). This supports the notion that stimulation strategies aimed at optimizing network efficiency may influence tolerance at high intensities (Robertson and Marino, 2016; De Wachter et al., 2021). Our previous research (Figure 1) has also found a significant correlation between the activation of the prefrontal and motor cortices and lower-limb performance during incremental loading to exhaustion (Liang, 2024). Building on these findings, multifocal tDCS (m-tDCS) targeting these key regions could potentially produce network-level modulation effects.

Therefore, this study tested whether m-tDCS targeting lower-limb-specific cortical areas could optimize late-stage performance and phase-specific muscle coordination during cycling via focusing on terminal-phase behavioral outcomes and on MCR and CAI across propulsion and pull phases as mechanistic endpoints. Two independent trials were conducted. Trial 1 tested the tolerability of the m-tDCS protocol, whereas Trial 2 evaluated its effects on lower-limb cycling endurance motor performance during an incremental loading cycling exercise using multimodal performance measures such as electromyography, physiological responses, and subjective effort ratings.

## 2 Methods

### 2.1 Participants

Sixty healthy young adults who met the eligibility criteria were recruited to participate in Trial 1 and Trial 2. The eligibility criteria were established based on Liang (2024).

Trial 1. A total of 15 healthy young adults voluntarily participated in the tolerance test for tailored multifocal stimulation (Table 1); to avoid participants guessing the experimental purpose and optimize blind effects, participants in Trial 1 were not allowed to take part in Trial 2.

**TABLE 1:** Participant demographics at baseline for Trial 1 and Trial 2.

Demographic	m-tDCS	Sham
Trial 1		
Sample	15	
Sex, n (%)		
Female	6 (40)	-
Male	9 (60)	-
Age, years	21.60 ± 2.91	-
Height, cm	163.90 ± 6.62	-
Weight, kg	53.50 ± 8.49	-
Body mass index, kg/m^2^	21.64 ± 0.07	-
Trial 2		
Sample	22	23
Sex, n (%)		
Female	10 (45)	11 (48)
Male	12 (55)	12 (52)
Age, years	22.18 ± 2.34	21.41 ± 3.16
Height, cm	170.82 ± 8.43	174.17 ± 7.39
Weight, kg	65.09 ± 18.08	71.46 ± 11.29
Body mass index, kg/m^2^	22.11 ± 4.95	23.47 ± 2.83

Trial 2. A total of 45 healthy young adults with the same characteristics participated in the tDCS and endurance exercise tests. Participants were randomly allocated to two groups to either receive one session of m-tDCS or one session of sham stimulation. Before trial testing, all participants had to pass a medical screening, were informed of the procedures, and provided informed written consent. The study was approved by the Ethics Committee of the Shanghai University of Sport under the registration number 102772023RT031, and its protocol adhered to the 1964 Declaration of Helsinki and its revisions.

### 2.2 Experiment protocol

Two trials were used to evaluate the m-tDCS effects on endurance capacity. Trial 1 was designed to verify the physical tolerance on tailored tDCS, whereas Trial 2, a randomized, sham-controlled, and double-blinded study, was designed to investigate the effects of m-tDCS on lower-limb cycling endurance motor performance.

During Trial 1, participants received 21 min of multifocal tDCS while sitting; the physical tolerance and side effects at different time points throughout the stimulation were monitored concurrently using the pain and side effect scales. During Trial 2, participants were asked to visit the laboratory on two consecutive days. The first visit was to investigate the baseline for maximum endurance capacity on the cycling ergometer. After 24 h, the participants visited the laboratory for the second visit. On the second visit, subjects first received either a 21-min m-tDCS or sham stimulation and then completed the incremental loading cycling immediately after stimulation, during which the physiological performance, psychological performance, and dominant muscle activity at termination phases were also measured to evaluate cycling endurance performance. To achieve the best motor performance, participants were verbally encouraged throughout the test.

### 2.3 Multifocal transcranial direct current stimulation

**FIGURE 2:**
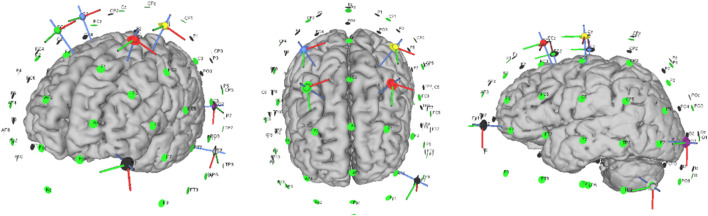
Electrode configurations of the stimulation protocol in this study.

The Neuroelectrics Starstim stimulator (Neuroelectrics, Barcelona, Spain) was used to deliver m-tDCS and sham stimulation through a set of seven circular Ag/AgCl electrodes with a surface area of 1.13 cm^2^. Based upon our previous findings (Liang et al., 2024), seven electrodes were placed in the C1, C2, FC1, FC2, AF7, P9, and P07 positions according to the 10/10 international EEG system, during which the participants received an anodal current over bilateral motor cortices (C1 and C2) and bilateral superior cortices (FC1 and FC2) and a cathodal current over the left lateral occipital cortex (P07 and P9) and left frontal pole (AF7) (Figure 2). The current intensity at each electrode was set to 1.053 mA from C1, 0.77 mA from C2, 1.221 mA from FC1, 0.954 mA from FC2, −1.016 mA from AF7, −1.430 mA from P9, and −1.552 mA from P07; the corresponding current densities were 0.93, 0.68, 1.08, 0.84, 0.90, 1.27, and 1.37 mA·cm^−2^, respectively. To mimic cutaneous sensations while maintaining blinding, the stimulator in the sham condition delivered a 30-s ramp-up immediately followed by a 30-s ramp-down to 0 mA, after which the current was set to zero for the remainder of the session. During stimulation, both the investigator and the participants were blinded to the type of stimulation the participants received.

### 2.4 Simulation of electric field characteristics for the stimulation protocol

**FIGURE 3:**
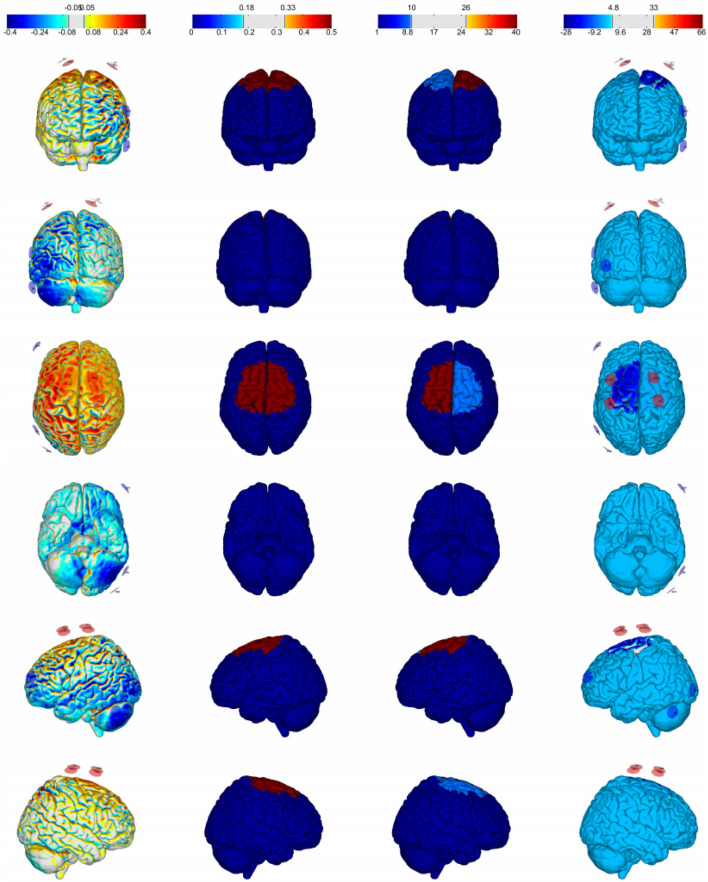
Distribution of the electric field of the stimulation protocol.

SimNIBS 4.0 with the algorithm and finite element model was used to calculate the electric field of m-tDCS protocol based on Ruffini et al. (2014). MRI data, the Colin 27, were segmented into the scalp, skull, cerebrospinal fluid, gray matter, and white matter. Conductivities of the anatomical tissues were set to σ-skin = 0.330 S/m; σ-bone = 0.008 S/m; σ-cerebrospinal fluid = 1.790 S/m; σ-gray matter = 0.400 S/m; and σ-white matter = 0.150 S/m. Electrodes were set up as cylinders with a radius of 10 mm and a thickness of 3 mm. Every tissue was regarded as isotropic and homogeneous. In order to best fit the spatial topography of the targeted brain regions, the target cortical solution was represented by the error relative to no intervention, a metric serving as a measure of quality of fit. The current intensity at each electrode was set up following the above m-tDCS protocol (Figure 3).

### 2.5 Tolerability and safety of the stimulation protocol

The pain perceived and side effects during stimulation were monitored to evaluate the tolerability and safety of m-tDCS. The participants were asked to report the subjective pain level and any of the adverse effects under the electrodes at the stimulation onset (30-s up); at 30 s, 1 min, 5 min, 10 min, 15 min, and 20 min; and at the end of stimulation (30-s down), respectively. The pain intensity was rated on an 11-point numerical rating scale (NRS) consisting of integers ranging from 0 to 10 (0: no pain; 3–7: moderate pain; and 7–10: worst pain possible). The tingling side effect under the electrodes was monitored, including headache, itching, burning sensation under electrodes, sleepiness, trouble concentrating, and acute mood change.

### 2.6 Incremental loading exercise protocol

The incremental loading exercise was adopted as the motor task to determine the dominant lower-limb maximum capacity of each participant. Participants had to cycle for 3 min without any load on a cycle ergometer (282E, Monark, Sweden), and subsequently completed the incremental cycling. During cycling, the incremental loading was increased by 20 W per minute for female participants and by 25 W per minute for male participants until voluntary exhaustion. During cycling, the criterion to stop the exercise was in keeping with Liang (2024). The cycling time and the peak power output (PPO) were recorded simultaneously. Based on a previous study, we divided the whole incremental loading exercise into four phases: 0%–50% PPO, 50%–75% PPO, 75%–85% PPO, and 85%–100% PPO (O’Bryan et al., 2014). The performance at 85%–100% PPO, corresponding to the maximum motor capacity, was utilized to evaluate effects of m-tDCS on lower-limb motor capacity via assessing time-to-exhaustion (TTE), work (W), average power (P), and revolutions per minute (RPM) throughout this phase.

### 2.7 Evaluation of physiological and psychological performances

The heart rate (HR), blood lactate level (ΔL), and rating of perceived effort (RPE) were monitored to assess physiological and psychological performances at the termination phase. HR was measured using a wireless heart-rate belt (Polar H10, Polar Electro Oy, Finland), with the sensor worn at the sternal position and the elastic band adjusted to ensure the constant positioning of the receiver during cycling. Furthermore, the blood lactate level (ΔL) was measured using the portable Lactate Scout 4 Solo analyzer (EKF Diagnostics, Germany) before and after the incremental loading exercise intervention. During testing, a second blood sample was taken at the right earlobe to avoid unnecessary distractions (e.g., sweat).

To assess the psychological performance related to subjective feelings of fatigue and loading (Borg, 1982), the 20-scale Borg rating of perceived effort was implemented to quantify exercise fatigue at each incremental loading; the collection time was consistent with the participants’ HR at that time, which was the last 10 s of each loading.

### 2.8 Evaluation of lower-limb muscle activity

Surface electromyography (sEMG) of the dominant lower limb as the key parameter was used to evaluate the effect of m-tDCS on lower-limb motor capacity, data for which were collected from eight active lower-limb muscles during cycling using an Ultium EMG system (Noraxon, United States) with a sampling frequency of 2,000 Hz. The monitored muscles included the rectus femoris (RF), vastus lateralis (VL), vastus medialis (VM), tibialis anterior (TA), long head of the biceps femoris (BF), semitendinosus (ST), medial gastrocnemius (GM), and lateral gastrocnemius (GL). For each loading phase of cycling, sEMG data were recorded starting from the 10th second of the loading phase and continued for a duration of 20 s, and the cycling period was divided into two phases: the propulsion and pull phases based on the recorded ankle joint motion. The sEMG of the dominant lower-limb muscle was analyzed for root mean square (RMS) amplitude, which was used to calculate the muscle contribution ratio (MCR) and co-activation index (CAI) of the knee joint at 85%–100% PPO, to further qualify the m-tDCS effects on lower-limb motor capacity.

### 2.9 Data processing

#### 2.9.1 Peak power output processing

The calculation of PPO adopted the following formulas that have been previously published in Jeukendrup et al. (1996):

$$\text{Male}:\;\text{PPO}=W_{\text{out}}+\left(\frac{t}{60}\times 25\right),$$

$$\text{Female}:\;\text{PPQ}=W_{\text{out}}+\left(\frac{t}{60}\times 20\right).$$

Here, W_out_ is the last loading value in watts (w) and t is the cycling lasting time during the last loading in seconds (s).

#### 2.9.2 Electromyography data processing

The sEMG data were processed using the Visual 3D™ software (C-Motion, United States). The specific processing steps for the sEMG signals are as follows: DC offset removal, band-pass filtering (10 Hz–400 Hz, second-order Butterworth filter), full-wave rectification, and moving average filtering (window width: 250 ms) to obtain the linear envelope of the EMG signal with a cutoff frequency of 6 Hz. RMS values for the propulsion and pull phases were calculated based on the motion trajectory of the ankle joint along the Z-axis, averaged over 10 consecutive cycling cycles. The RMS values were then normalized using the maximum value from the maximal voluntary contraction (MVC). Finally, the data from the 10 cycling cycles were averaged to provide the final RMS value for the statistical analysis of the muscle activity level, the muscle contribution ratio, and CAI of the knee joint. The formulas for MCR and CAI of the knee joint are as follows:

The MCR was calculated to assess the relative contribution of each of the eight monitored muscles to the overall muscle activity during cycling. The MCR was defined as

$${MCR}_{i}\left(\%\right)=\frac{{RMS}_{i}}{\sum_{i=1}^{n=8}{RMS}_{i}}\times 100\%,$$

where RMS_*i*_ refers to the RMS value of the EMG signal for the *i*th muscle, representing the level of muscle activation during cycling.

The denominator represents the sum of the RMS values of all eight monitored muscles, which gives the total muscle activity across all muscles.

The CAI was calculated to assess the level of co-activation between the agonist and antagonist muscles during knee flexion and extension. The CAI was defined as

$$CAI=\frac{RMS\;of\;Antagonist}{RMS\;of\;Agonist+RMS\;of\;Antagonist},$$

where “RMS of Agonist” refers to the RMS value of the EMG signal of the agonist muscle (e.g., quadriceps for knee extension) and “RMS of Antagonist” refers to the RMS value of the EMG signal of the antagonist muscle (e.g., hamstrings for knee extension).

The CAI ranges from 0 to 1, where a value close to 0 indicates minimal co-activation of the antagonist muscle, suggesting that the agonist muscle primarily controls the movement. A value approaching 1 indicates significant co-activation of both muscles, which may reflect the need for joint stability or reduced movement amplitude during the specific phase of motion.

### 2.10 Statistical analysis

All collected data were expressed as mean ± standard error (M ± SEM).

Trial 1. Mauchly’s test was used to assess the sphericity of the repeated-measures factor. If the sphericity assumption was violated, the Greenhouse–Geisser correction was applied to adjust the degrees of freedom. A one-way repeated-measures analysis of variance (ANOVA) was conducted to analyze the NRS scores at different time points during m-tDCS. Post-hoc pairwise comparisons were performed using the Bonferroni correction. The effect size was calculated using partial eta-squared $\eta_{p}^{2}$ with the following criteria: 0.01–0.09 for a low effect, 0.09–0.25 for a medium effect, and >0.25 for a high effect. The side effects related to the stimulation protocol that were reported by the participants were summarized descriptively.

Trial 2. Student’s unpaired t-test was used to compare the baseline characteristics (age, body mass index, lower-limb motor capacity, HR, RPE, and ΔL) between the two intervention groups (m-tDCS and sham) to ensure comparability prior to stimulation. To evaluate the effect of m-tDCS on lower-limb motor capacity, a mixed-model ANOVA was conducted, with time (pre-stimulation and post-stimulation) as the within-subjects factor and group (m-tDCS and sham) as the between-subjects factor. The following outcome measures collected at 85%–100% PPO were analyzed: time to TTE, W, P, RPM, HR, RPE, ΔL, MRC, and CAI. When significant interaction effects were detected, Bonferroni-corrected post-hoc pairwise comparisons were performed. For main effects of time or protocol, post-hoc analysis was conducted using paired Student’s t-test (for time) or unpaired Student’s t-test (for group). If significant baseline differences were observed between groups, further analysis was performed using unpaired Student’s t-test to compare the change in variables from pre- to post-stimulation between the two groups. Effect sizes were calculated as partial eta-squared $\eta_{p}^{2}$ , with thresholds as described above.

## 3 Results

### 3.1 Testing the performance of the stimulation protocol’s tolerability and side effects

Nevertheless, the pain levels decreased progressively as the stimulation continued (see Table 2). A significant change in pain levels was observed throughout the stimulation period, with a main effect of time. No participant reported extreme pain ratings, and there were no dropouts in the experiment. A total of 73.3% of the participants experienced a tingling sensation under the electrodes during m-tDCS, with 53.3% reporting itching, 40% reporting a burning sensation, and 6.67% reporting drowsiness.

**TABLE 2:** Subjective pain perception during the stimulation process.

Phase	Pain score	F	*p*	$\eta_{p}^{2}$
30-s up	3.93 ± 0.55^a^	11.14	0.001	0.91
30 s	4.80 ± 0.45^b^			
1 min	4.53 ± 0.55^c^			
5 min	3.33 ± 0.49^d^			
10 min	2.4 ± 0.57			
15 min	1.79 ± 0.33^a,b,d^			
20 min	1.71 ± 0.32^b,c^			
30-s down	2.07 ± 0.33^b,c^			

*a* indicates that the pain level at 30 s was significantly different from that at 15 min; *b* indicates that the pain level at 30 s was significantly different from those at 15 min, 20 min, and 30-s down; *c* indicates that the pain perception at 1 min was significantly different from those at 15 min, 20 min, and 30-s down; and *d* indicates that the pain level at 5 min was significantly different from that at 15 min; *p* < 0.05.

### 3.2 Effects of m-tDCS on motor performance

#### 3.2.1 Comparison of the participant-based characteristics and motor capacity

All participants successfully completed the incremental exercise test, which enabled a valid comparison of motor capacity. No significant differences in participant demographics and lower-limb maximum motor capacity were found between the m-tDCS and sham groups (p > 0.05; see Table 3).

**TABLE 3:** Comparison of the participant demographics and maximum motor capacity.

Demographic	m-tDCS	Sham	*t*	*p*
Age, years	22.18 ± 2.34	21.41 ± 3.16	0.92	0.362
Height, cm	170.82 ± 8.43	174.17 ± 7.39	−1.40	0.168
Weight, kg	65.09 ± 18.08	71.46 ± 11.29	−1.40	0.168
Body mass index, kg/m^2^	22.11 ± 4.95	23.47 ± 2.83	−1.12	0.270
TTE, s	759.77 ± 22.76	715.55 ± 20.98	−1.42	0.161
PPO, w	232.98 ± 12.08	215.3 ± 11.63	−1.08	0.284
Work, J	26.54 ± 2.30	23.22 ± 2.58	−0.73	0.468
Power, W	160.49 ± 10.73	160.02 ± 17.54	−0.02	0.982
HR, bpm	173.81 ± 3.23	170.6 ± 2.27	−0.81	0.421
RPE	18.04 ± 1.38	18 ± 1.27	−0.11	0.916
RPM, r/min	70.69 ± 6.01	67.47 ± 5.02	−1.93	0.061
ΔL, mol/L	11.03 ± 0.46	10.33 ± 0.45	−1.03	0.307

#### 3.2.2 Cycling endurance performance at 85%–100% PPO

Participants completed the test to their maximum motor capacity. No participants reported any sports injury during cycling.

##### 3.2.2.1 Motor performance of the lower limb

**FIGURE 4:**
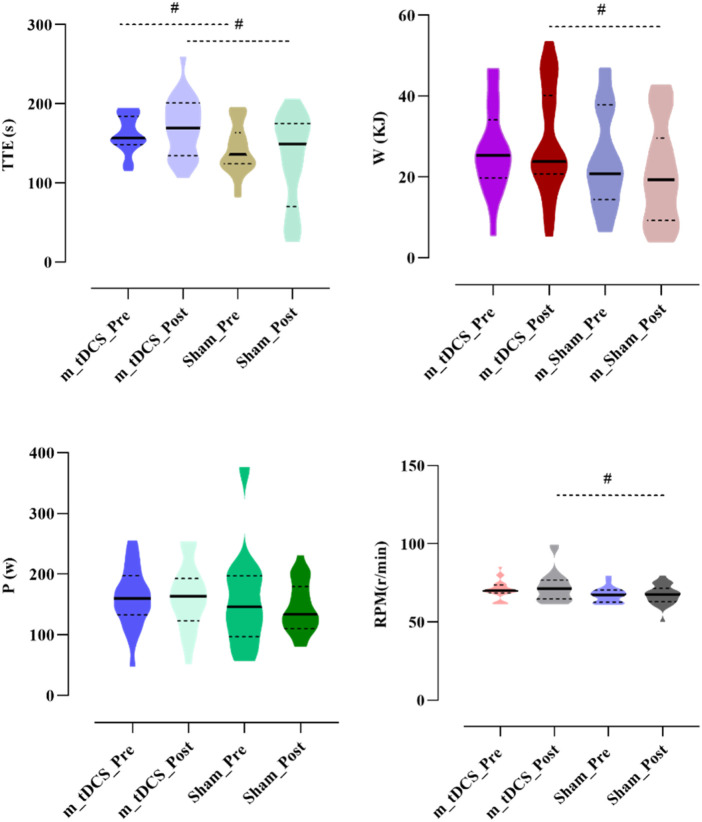
Effects of m-tDCS on lower-limb motor performance at 85%–100% PPO. TTE indicates cycling time; W indicates the work; P indicates the power output, RPM indicates revolutions per minute. # indicates the significant differences between groups: p < 0.05.

A significant stimulation protocol × time interaction was found for W (p < 0.05) (Table 4) (Figure 4). A between-group comparison at baseline showed no significant difference (F = 0.92, p = 0.342), whereas post-stimulation, W of the m-tDCS group was significantly greater than that of the sham group (F = 4.81, p = 0.034).

**TABLE 4:** Motor performance before and after stimulation at 85%–100% PPO.

Index	m-tDCS		Sham		F	*p*	$\eta_{p}^{2}$
	Before	After	Before	After			
Motor performance							
TTE (s)	161.10 ± 5.04	170.72 ± 8.17	142.88 ± 6.24	129.65 ± 11.89	F_time_ = 0.09	*p*_time_ = 0.764	0.01
					F_condition_ = 8.54	*p*_condition_ = 0.006	0.17
					F_time*condition_ = 3.67	*p*_time*condition_ = 0.062	0.08
W (KJ)	26.54 ± 2.30	28.68 ± 2.88	23.22 ± 2.58	20.09 ± 2.66	F_time_ = 0.17	*p*_time_ = 0.684	0.01
					F_condition_ = 2.91	*p*_condition_ = 0.095	0.50
					F_time*condition_ = 4.81	*p*_time*condition_ = 0.034	0.10
P (w)	160.49 ± 10.73	161.44 ± 11.56	160.02 ± 17.54	146.06 ± 8.80	F_time_ = 0.48	*p*_time_ = 0.494	0.01
					F_condition_ = 0.27	*p*_condition_ = 0.605	0.01
					F_time*condition_ = 0.63	*p*_time*condition_ = 0.434	0.01
RPM (r/min))	70.69 ± 1.28	72.66 ± 2.18	67.47 ± 1.07	67.33 ± 1.41	F_time_ = 0.74	*p*_time_ = 0.396	0.02
					F_condition_ = 5.04	*p*_condition_ = 0.030	0.11
					F_time*condition_ = 0.972	*p*_time*condition_ = 0.330	0.02
Physiological performance							
HR (bpm)	173.81 ± 3.23	168.03 ± 2.81	170.60 ± 2.27	167.91 ± 2.74	F_time_ = 11.72	*p*_time_ = 0.001	0.22
					F_condition_ = 0.20	*p*_condition_ = 0.659	0.01
					F_time*condition_ = 1.57	*p*_time*condition_ = 0.218	0.04
ΔL (mol/L)	11.03 ± 0.46	10.7 ± 0.43	10.33 ± 0.45	10.76 ± 0.42	F_time_ = 0.03	*p*_time_ = 0.860	0.01
					F_condition_ = 0.36	*p*_condition_ = 0.549	0.01
					F_time*condition_ = 1.44	*p*_time*condition_ = 0.236	0.03
Psychological performance							
RPE	18.04 ± 0.29	18.32 ± 0.32	18 ± 0.27	18.47 ± 0.31	F_time_ = 6.15	*p*_time_ = 0.017	0.13
					F_condition_ = 0.02	*p*_condition_ = 0.894	0.01
					F_time*condition_ = 0.40	*p*_time*condition_ = 0.532	0.01

TTE indicates cycling time; W indicates the work; P indicates the power output; HR indicates the heart rate; RPE indicates the rate of perception effort; RPM indicates revolutions per minute; ΔL indicates blood lactate level.

A significant main effect of the stimulation protocol was observed for TTE (p < 0.05) (Table 4) (Figure 4). TTE was significantly higher in the m-tDCS group than in the sham group at both pre- (t = −2.26, p = 0.029) and post-stimulation (t = −2.82, p = 0.007). However, there was no significant change in TTE between groups (t = −1.92, p = 0.062).

A significant main effect of the stimulation protocol was also found for RPM (p < 0.05) (Table 4) (Figure 4). At baseline, no difference in RPM was recorded between m-tDCS and sham groups (t = −1.93, p = 0.061); however, after stimulation, RPM was significantly higher in the m-tDCS group than in the sham group (t = −2.22, p = 0.032).

No significant main effects of stimulation protocol or stimulation time, nor their interaction, were observed for P (p > 0.05).

##### 3.2.2.2 Physiological performance

**FIGURE 5:**
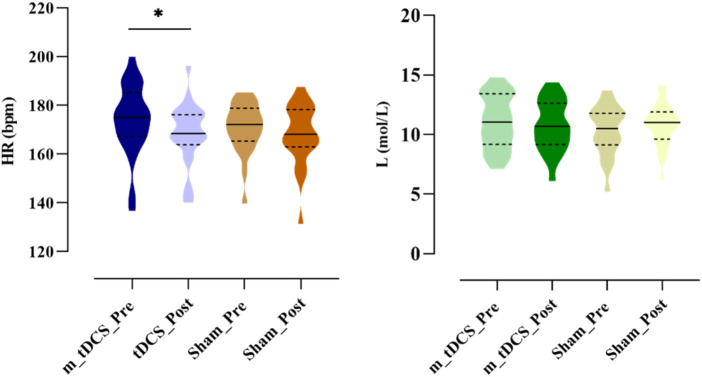
Effects of m-tDCS on the physiological performance at 85%–100% PPO. HR indicates the heart rate; ΔL indicates the blood lactate level. ^*^ indicates the significant differences between pre- and post-intervention: p < 0.05.

A significant main effect of stimulation time was found for HR (p < 0.05) (Table 3) (Figure 5). Post-hoc analysis indicated that HR was significantly reduced after m-tDCS (t = 3.65, p = 0.001), whereas no significant change was observed in the sham group (t = 1.41, p = 0.172). No significant main effects of stimulation protocol or stimulation time, nor their interaction, were detected for ΔL (p > 0.05).

##### 3.2.2.3 Psychological performance

**FIGURE 6:**
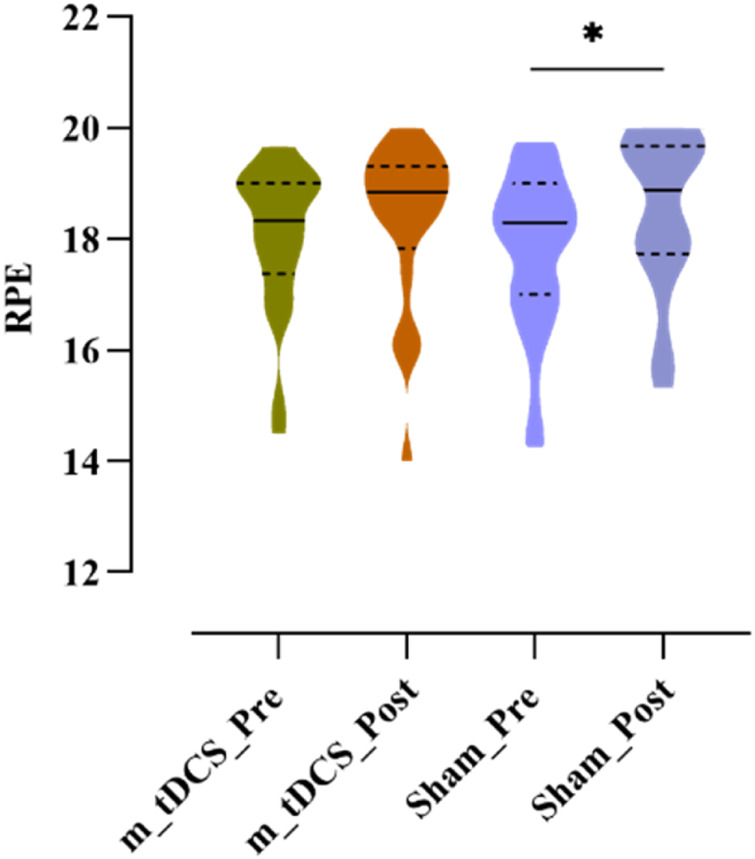
Effects of m-tDCS on the psychological performance at 85%–100% PPO. RPE indicates the rate of perception effort. ^*^ indicates the significant differences between pre- and post-intervention: p < 0.05.

Analysis of RPE showed a significant main effect of stimulation time (p < 0.05) (Table 3) (Figure 6). Post-hoc analysis revealed that RPE significantly increased after sham stimulation (t = −2.09, p = 0.049), whereas no significant change was observed following tDCS (t = −1.39, p = 0.180).

#### 3.2.3 Contribution of the dominant lower-limb muscle before and after stimulation

##### 3.2.3.1 Performance of the lower-limb muscle in the propulsion phase

**FIGURE 7:**
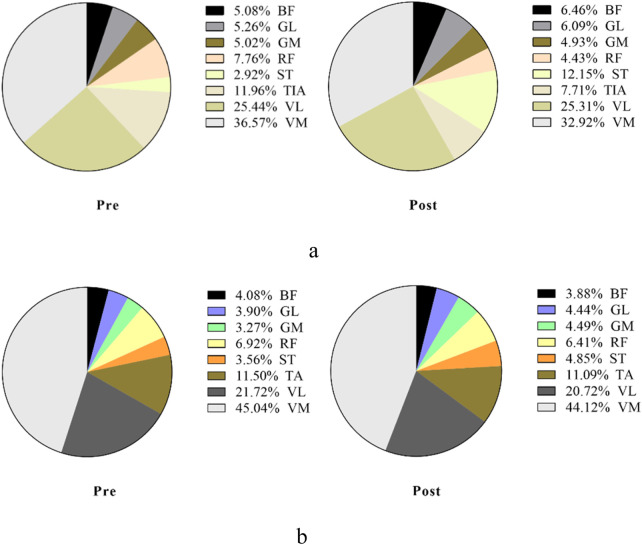
Overall view of MCR changes at different interventions during the propulsion phase at 85%–100% PPO. MCR indicates muscle contribution ratio; RF indicates rectus femoris; VL indicates vastus lateralis; VM indicates vastus medialis; BF indicates biceps femoris, long head; ST indicates semitendinosus; GM indicates medial gastrocnemius; GL indicates lateral gastrocnemius. **(a)** MCR changes in the sham group. **(b)** MCR changes in the m-tDCS group.

**FIGURE 8:**
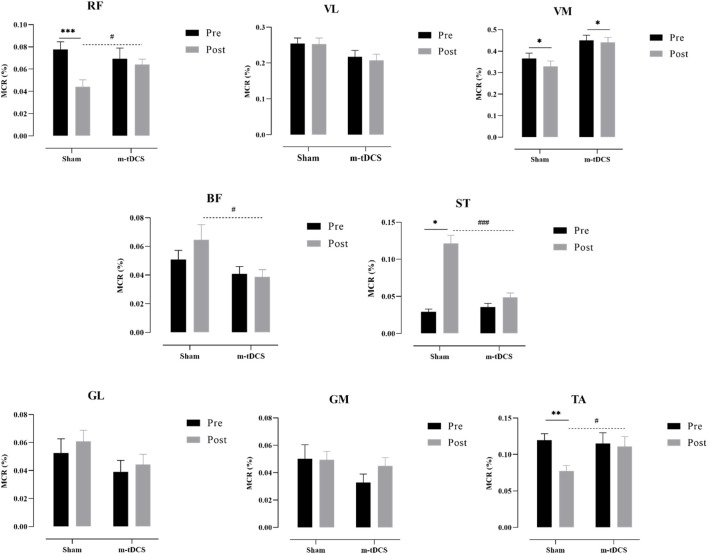
Performance of the muscle contribution ratio of the lower-limb muscle in the propulsion phase. MCR indicates the muscle contribution ratio; RF indicates rectus femoris; VL indicates vastus lateralis; VM indicates vastus medialis; BF indicates biceps femoris, long head; ST indicates semitendinosus; GM indicates medial gastrocnemius; GL indicates lateral gastrocnemius. The asterisk indicates the significant difference between pre- and post-intervention: ^*^ represents p < 0.05, ^**^ represents p < 0.01, and ^***^ represents p < 0.001; the well number indicates the significant difference between groups: # represents p < 0.05, and ### represents p < 0.001.

Figures 7, 8 show the overall view of MCR performance in the propulsion phase. The interaction between stimulation time and stimulation protocol significantly affected RF-MCR (p < 0.05). Within-group analysis revealed that MCR significantly decreased after sham stimulation (t = 4.38, p < 0.001), whereas no significant change was observed following m-tDCS (t = 0.67, p = 0.513). Between-group analysis showed no significant difference in MCR between m-tDCS and sham groups at baseline (t = 0.64, p = 0.528); however, after stimulation, MCR of the m-tDCS group was significantly higher than that of the sham group (t = −2.59, p = 0.013). The interaction between stimulation time and protocol was also significant for ST-MCR (p < 0.05). Within-group analysis showed a significant increase in MCR following sham stimulation (t = −1.95, p = 0.066), whereas no significant change occurred after m-tDCS (t = 0.67, p = 0.513). Between-group analysis revealed no significant difference between m-tDCS and sham groups at baseline (t = −0.84, p = 0.404), but MCR of the m-tDCS group was significantly lower than that in the sham group after stimulation (t = 5.77, p < 0.001). For TA, the interaction between the stimulation time and protocol was also significant (p < 0.05). Within-group analysis revealed a significant decrease in MCR after sham stimulation (t = 3.83, p = 0.001), whereas no significant change occurred after m-tDCS (t = 0.18, p = 0.861). Between-group analysis showed no significant difference in MCR between m-tDCS and sham groups at baseline (t = 0.38, p = 0.704), but MCR of the m-tDCS group was significantly higher than that of the sham group after stimulation (t = −2.15, p = 0.038).

There was a significant main effect of the stimulation protocol on BF-MCR (p < 0.05). No significant difference was found in MCR between m-tDCS and sham groups at baseline (t = 1.17, p = 0.247), but MCR of the m-tDCS group was significantly lower than that of the sham group after stimulation (t = 2.14, p = 0.039). A significant main effect of the stimulation protocol was also observed for VM-MCR (p < 0.05). The MCR of the m-tDCS group was significantly higher than that of the sham group at both baseline (t = −2.44, p = 0.019) and post-stimulation (t = −3.40, p = 0.02). Further analysis of the change in MCR showed no significant difference between the two intervention groups (t = −0.80, p = 0.428).

No significant interaction between stimulation time and protocol was found for GL-, GM-, or VL-MCR (p > 0.05), and no significant main effects of time or protocol were observed for these muscle groups (p > 0.05).

##### 3.2.3.2 Performance of the lower-limb muscle in the pull phase

**FIGURE 9:**
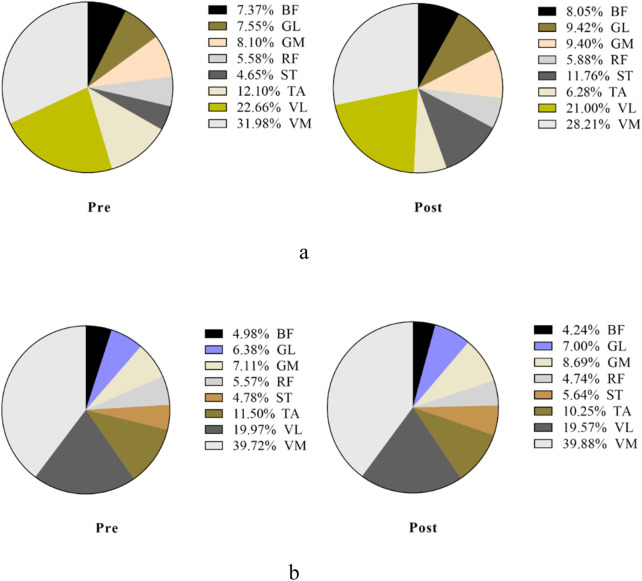
Overall view of MCR changes at different interventions during the pull phase at 85%–100% PPO. MCR indicates the muscle contribution ratio; RF indicates rectus femoris; VL indicates vastus lateralis; VM indicates vastus medialis; BF indicates biceps femoris, long head; ST indicates semitendinosus; GM indicates medial gastrocnemius; GL indicates lateral gastrocnemius. **(a)** MCR changes in the sham group. **(b)** MCR changes in the m-tDCS group.

**FIGURE 10:**
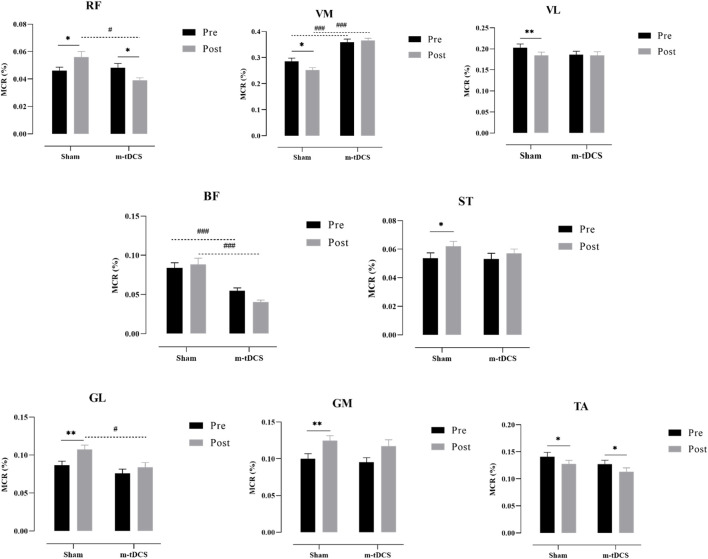
Performance of the muscle contribution ratio of the lower-limb muscle in the pull phase. MCR indicates muscle contribution ratio; RF is rectus femoris; VL indicates vastus lateralis; VM indicates vastus medialis; BF indicates biceps femoris, long head; ST indicates semitendinosus; GM indicates medial gastrocnemius; GL indicates lateral gastrocnemius. The asterisk indicates the significant difference between pre- and post-intervention: ^*^ represents p < 0.05, ^**^ represents p < 0.01, and ^***^ represents p < 0.001; the well number indicates the significant difference between groups: # represents p < 0.05, and ### represents p < 0.001.

Figures 9, 10 show the overall view of MCR performance in the pull phase. The interaction between stimulation protocol and stimulation time had a significant effect on RF-MCR (p < 0.05) (Figure 9). Within-group analysis revealed that RF-MCR was significantly increased after sham stimulation (t = −2.09, p = 0.040), whereas it significantly decreased following m-tDCS (t = 3.59, p = 0.01). Between-group analysis showed no significant difference in RF-MCR between m-tDCS and sham groups at baseline (t = 0.35, p = 0.726), but MCR was significantly lower in the m-tDCS group than in the sham group after stimulation (t = 3.90, p < 0.01). The interaction between stimulation protocol and stimulation time was also significant for the VM (p < 0.05). Within-group analysis showed that VM-MCR significantly decreased after sham stimulation (t = 2.78, p = 0.007), whereas no significant change was observed after m-tDCS (t = −0.71, p = 0.479). Between-group analysis indicated that VM-MCR was significantly higher in the m-tDCS group than in the sham group at both baseline (t = −4.18, p < 0.001) and post-stimulation (t = −8.67, p < 0.001). Further analysis of the change in MCR revealed that the reduction in MCR after sham stimulation was significantly greater than that after m-tDCS (t = −2.65, p = 0.009).

A significant main effect of the stimulation protocol was found for the BF (p < 0.05). BF-MCR was significantly higher in the m-tDCS group than in the sham group at both baseline (t = 3.87, p < 0.001) and post-stimulation (t = 5.63, p < 0.001). However, there was no significant difference in MCR between m-tDCS and sham groups (t = 1.78, p = 0.076). A significant main effect of stimulation protocol was also observed for the GL (p < 0.05). At baseline, there was no significant difference in MCR between m-tDCS and sham groups (t = 1.40, p = 0.164). However, after stimulation, MCR was significantly lower in the m-tDCS group than in the sham group (t = 2.83, p = 0.005).

Significant main effects of time were observed for VL-, ST-, TA-, GM-, and GL-MCR (p < 0.05). Specifically, within-group analysis showed that VL-MCR significantly decreased after sham stimulation (t = 2.67, p = 0.009), but no significant change was found after m-tDCS (t = 0.30, p = 0.762). The ST-MCR increased significantly after sham stimulation (t = −2.00, p = 0.049), whereas no significant change was found following m-tDCS (t = −1.15, p = 0.255). Both sham and m-tDCS groups showed significant reductions in TA-MCR (sham: t = 2.30, p = 0.024; m-tDCS: t = 2.20, p = 0.031). The GM-MCR increased significantly in both sham and m-tDCS groups (sham: t = −2.96, p = 0.004; m-tDCS: t = −4.24, p < 0.001). Finally, the GL-MCR increased significantly after sham stimulation (t = −3.44, p = 0.001), but no significant change was observed following m-tDCS (t = −1.78, p = 0.079).

#### 3.2.4 Performance comparison of antagonist and agonist muscles in the knee joint before and after stimulation

**FIGURE 11:**
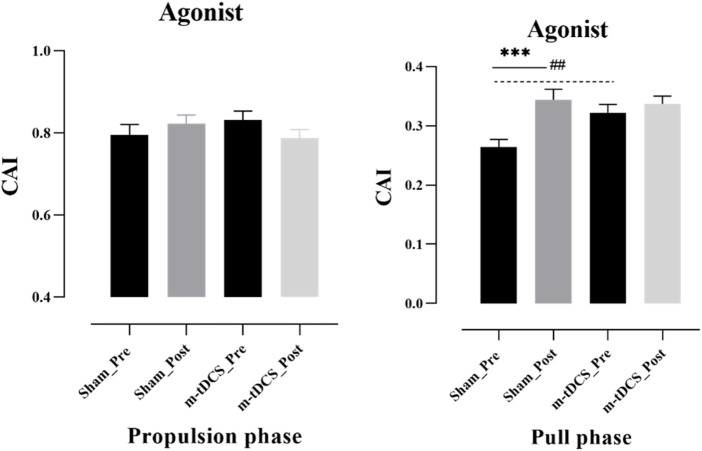
Co-activation index of agonist muscles around the knee at 85%–100% PPO. The asterisk indicates the significant difference between pre- and post-intervention: ^***^ represents p < 0.001; the well number indicates the significant difference between groups: ## represents p < 0.01.

Based on the characteristics of the movement cycle, the muscles monitored in this study were categorized as follows: during the propulsion phase, the RF, VL, and VM were classified as agonist muscles, whereas the BF, ST, GM, and GL acted as antagonist muscles. Analysis of the CAI for active muscles during the propulsion phase revealed no significant interaction effects between m-tDCS and sham groups (p > 0.05), and there were no significant main effects of stimulation protocol or stimulation time (p > 0.05) (Figure 11).

In contrast to the propulsion phase, the BF, ST, GM, and GL acted as agonist muscles, whereas the RF, VL, and VM were classified as antagonist muscles during the pull phase. Analysis of the CAI for agonist muscles during the pull phase revealed a significant interaction effect between stimulation time and protocol (p < 0.05). Within-group analysis showed a significant increase in CAI after sham stimulation compared to baseline (t = −5.49, p < 0.001), whereas no significant difference was observed before and after m-tDCS (t = −1.45, p = 0.152). Between-group analysis revealed that the CAI in the m-tDCS group was significantly higher than that in the sham group at baseline (t = −2.97, p = 0.003), but no significant difference was found between the two groups after stimulation (t = 0.32, p = 0.748). Further analysis of the changes in CAI between sham stimulation and m-tDCS revealed that the increase in CAI in the sham group was significantly greater than that in the m-tDCS group (t = 3.61, p < 0.001).

## 4 Discussion

This study aimed to evaluate the effects of m-tDCS targeting lower-limb-specific cortical areas on cycling endurance motor performance in healthy adults. The findings provide compelling evidence that m-tDCS is a safe and non-invasive brain stimulation technique that can significantly enhance lower-limb endurance capacity. This improvement is accompanied by changes in lower-limb muscle activity and coordination, suggesting that m-tDCS optimizes endurance performance by modulating cortical excitability. Additionally, significant differences in the RPM were observed during the high-intensity phase, indicating enhanced motor control and coordination post-stimulation.

The multifocal montage is designed to engage a distributed motor network, potentially increasing focality at cortical targets and improving current steering relative to single-site approaches (Dmochowski et al., 2011). Within this framework, the present propulsion-specific maintenance of quadriceps MCR and the pull-phase restraint of antagonistic co-activation (stable CAI) align with enhanced neural efficiency—i.e., a more economical recruitment of task-relevant synergies and suppression of non-task-relevant activity (Falconer and Winter, 1985; Li et al., 2020). Because P and ΔL were unchanged, the performance pattern (higher W at post-stimulation vs. sham and higher RPM) is better explained by coordination-driven endurance support than by increased maximal power output or altered metabolic accumulation. Such synergy-level modulation aligns with prior reports that tDCS can facilitate endurance-type outcomes without necessarily increasing peak strength (Vitor-Costa et al., 2015; Angius et al., 2016; Angius et al., 2018; Alix-Fages et al., 2019; Chinzara et al., 2022).

A substantial body of work indicates that anodal or extracephalic tDCS may extend time-to-task failure and attenuate perceived exertion under certain protocols (Vitor-Costa et al., 2015; Angius et al., 2016; Angius et al., 2018). Meta-analytic summaries suggest small-to-moderate, parameter-dependent effects on endurance with mixed evidence for strength and power (Alix-Fages et al., 2019; Chinzara et al., 2022). The current findings refine this picture in two ways: (i) by using a multifocal montage that likely acts on a broader motor network (Dmochowski et al., 2011) and (ii) by linking behavioral outcomes to phase-dependent adjustments in MCR/CAI. Rather than a generalized boost in the maximal output, our results highlight preservation of terminal-phase work and cadence via coordination tuning—a distinction with practical relevance for a prolonged or fatiguing task. The significant changes in MCR and CAI observed in this study provide objective, physiological evidence of how m-tDCS modulates muscle activation patterns to enhance endurance performance.

Compared with previous studies, this study also contributes to the growing body of literature on tDCS by providing new insights into its effects on lower-limb motor performance, particularly endurance (Vitor-Costa et al., 2015; Angius et al., 2016; Angius et al., 2018; Alix-Fages et al., 2019; Chinzara et al., 2022). The changes in muscle coordination, as reflected by MCR and CAI, offer a novel mechanism through which m-tDCS enhances motor performance (Falconer and Winter, 1985; Li et al., 2020; Dmochowski et al., 2011). These findings support the idea that tDCS, especially multifocal stimulation, can improve neural efficiency, muscle activation, and coordination, leading to enhanced endurance (Dmochowski et al., 2011; Angius et al., 2018; Chinzara et al., 2022; Pilloni et al., 2020). From a theoretical standpoint, these results deepen our understanding of how brain stimulation affects the neural networks responsible for motor control and endurance (Dmochowski et al., 2011; Chinzara et al., 2022). The modulation of muscle coordination and the efficiency of muscle activation by m-tDCS could represent a key mechanism underlying the observed endurance improvements (Dmochowski et al., 2011; Falconer and Winter, 1985; Li et al., 2020; Alix-Fages et al., 2019).

Practically, these findings have significant implications for both sports performance and rehabilitation. The ability of m-tDCS to improve endurance performance and muscle coordination makes it a valuable tool for athletes in endurance sports. By enhancing neuromuscular coordination and efficiency, m-tDCS could help athletes optimize performance during prolonged physical exertion. In rehabilitation, m-tDCS could be used to assist individuals with motor impairments in improving muscle coordination and endurance (Pilloni et al., 2020; Li et al., 2020). Given the observed changes in MCR and CAI, m-tDCS may help enhance neuromuscular function in clinical populations, particularly those recovering from neurological disorders such as stroke or multiple sclerosis (Pilloni et al., 2020; Li et al., 2020). Therefore, based on our findings and from an applied standpoint, we suggest that m-tDCS is most likely to be effective when paired with task engagement. Specifically, in athletes, a single pre-task m-tDCS session is feasible to deliver immediately before high-intensity cycling as a practical use case, which may help preserve late-stage endurance by supporting phase-specific neuromuscular coordination. In rehabilitation, brief pre-therapy m-tDCS paired with task-specific practice may improve tolerance and neuromuscular efficiency (Pilloni et al., 2020; Li et al., 2020). Importantly, tDCS after-effects begin to develop during stimulation and usually persist for approximately 30 min–60 min—sometimes up to 90 min—showing a short onset and gradual decline. Consequently, more durable improvements likely require multi-session protocols.

Despite the valuable insights provided by this study, several limitations should be considered. The sample size was relatively small, and the study focused only on healthy young adults. Future research should involve larger and more diverse populations, including older adults and individuals with neurological conditions, to assess the generalizability of these findings. Moreover, although this study focused on endurance performance, future research should explore the effects of m-tDCS on other motor abilities, such as strength and coordination, to gain a broader understanding of its effects on motor function (Alix-Fages et al., 2019; Chinzara et al., 2022). Additionally, this study did not include long-term follow-up to assess the durability of the observed effects. Future studies should incorporate follow-up assessments to determine whether the performance improvements persist over time (Pilloni et al., 2020). Furthermore, the neural mechanisms underlying the effects of m-tDCS on muscle coordination remain to be elucidated. Although the results suggest changes in muscle coordination via increased MCR and reduced CAI, further investigation using neuroimaging techniques such as functional MRI or electroencephalography could provide more detailed insights into the brain activity changes that accompany these improvements in motor performance (Dmochowski et al., 2011). Finally, it is theorized that m-tDCS offers improved focality and intensity compared to the classical single-site tDCS approach; however, we did not conduct a direct comparison between these two techniques in our study. This comparison could further clarify the distinct advantages and potential differences in their effects on performance. We acknowledge the importance of this issue, and thus future research should explore and compare the effects of both techniques to provide a more comprehensive understanding of their relative efficacy.

## 5 Conclusion

m-tDCS, delivered with a multifocal montage, did not increase peak mechanical capacity but helped preserve late-stage work and cadence during high-intensity cycling, accompanied by phase-specific optimization of muscle coordination—maintenance of propulsion-relevant contributions and pull-phase restraint of antagonistic co-activation. These findings refine the view of the effects of tDCS on endurance: coordination-centric, network-level efficiency rather than a uniform boost in the output of motor capacity.

## Data Availability

The original contributions presented in the study are included in the article/supplementary material; further inquiries can be directed to the corresponding authors.
